# Risk of intracranial hemorrhage associated with autosomal dominant polycystic kidney disease in patients with end stage renal disease

**DOI:** 10.1186/1471-2369-15-39

**Published:** 2014-02-26

**Authors:** David J Yoo, Lawrence Agodoa, Christina M Yuan, Kevin C Abbott, Robert Nee

**Affiliations:** 1 Nephrology, Walter Reed National Military Medical Center, 8901 Wisconsin Ave, Bethesda, MD, USA; 2 NIDDK, National Institutes of Health, Bethesda, MD, USA

**Keywords:** Intracranial hemorrhage, Intracranial aneurysm, Autosomal dominant polycystic kidney disease, Stroke, Dialysis, USRDS, Competing risk

## Abstract

**Background:**

An analysis of intracranial hemorrhage (ICH) in a national sample of autosomal dominant polycystic kidney disease (ADPKD) patients receiving long-term dialysis has not been reported. It is often assumed that patients with ADPKD are not at increased risk of ICH after starting dialysis. We hypothesized that patients with ADPKD would have a higher subsequent risk of ICH even after the start of chronic dialysis.

**Methods:**

Retrospective cohort study of Medicare primary patients with and without ADPKD in the United States Renal Data System (USRDS), initiated on chronic dialysis or transplanted between 1 January 1999 and 3 July 2009, and followed until 31 December 2009. Covariates included age, gender, race, prior stroke, diabetes mellitus, dialysis modality, body mass index, serum albumin and other co-morbid conditions from the Medical Evidence Form. Primary outcome was ICH, based on inpatient and outpatient Medicare claims, and all-cause mortality. Kaplan-Meier analysis was used for unadjusted assessment of time to events. Cox regression was used for assessment of factors associated with ICH and mortality. We performed competing risk regression using kidney transplant and death as competing risks. Kidney transplant was also modeled as a time-dependent covariate in Cox regression.

**Results:**

Competing risk regression demonstrated that ADPKD had a subhazard ratio 2.97 for ICH (95% CI 2.27-3.89). Adjusted Cox analysis showed that ADPKD patients had an AHR for death of 0.59 vs. non-ADPKD patients (95% CI 0.57-0.61).

**Conclusions:**

ADPKD is a significant risk factor for ICH among patients on maintenance dialysis. Our Medicare primary cohort was older than in previous studies of intracranial aneurysm rupture among ADPKD patients. There are also limitations inherent to using the USRDS database.

## Background

Autosomal dominant polycystic kidney disease (ADPKD) is one of the most common single gene hereditary disorders, occurring in about 1 in every 400–1000 live births [1-3]. It is associated with renal, hepatic, and pancreatic cysts, valvular heart disease, diverticular disease, and intracranial aneurysms [4]. The genes responsible, PKD1 and PKD2, respectively, appear to have equivalent expression of intracranial aneurysms and play a direct pathogenetic role in aneurysm formation, [5,6]. A ruptured intracranial aneurysm is a potentially life-threatening complication of ADPKD, associated with considerable morbidity and mortality [7-10]. Intracranial aneurysm prevalence in ADPKD ranges from 4% to as high as 41% with a positive family history, increasing with age; rates per person-years are not available [2,11-14]. Aneurysmal subarachnoid hemorrhage (SAH) has a 30-day mortality rate of 45%. The survivors of these strokes have about a 30% chance of moderate or severe disability [15]. In comparison, in the general U.S. population the incidence of subarachnoid hemorrhage is 9–20 per 100,000 person-years (PY), with 30-day mortality of 35%-52%, [16,17]. The overall total intracranial hemorrhage (ICH) incidence in the U.S. is 24.6 per 100,000 PY [18]. In the general population, risk factors for ICH include male gender, older age, Asian ethnicity [19], and in the U.S., African American (AA) ethnicity [20]. The most important etiologies for ICH in the general population are hypertension (usually leading to hemorrhage in deep structures) and cerebral amyloid angiopathy (CAA) [21]. CAA is less likely related to blood pressure but more with older age, and associated ICH tends to occur in lobar regions [22]. Chief among genetic factors contributing to ICH is apolipoprotein E (APOE) gene and ɛ2 and ɛ4 alleles [23]. ADPKD is prominent among genetic contributors to SAH, along with first degree family history, Marfan’s syndrome and Ehlers-Danlos syndrome [16].

Patients with end-stage renal disease (ESRD) may be at increased risk of hemorrhagic stroke, due to hypertension, bleeding diatheses, and routine administration of heparin during hemodialysis. A recent USRDS study by Seliger reported an incidence ICH in hemodialysis patients was about 5 per 1000 PY [18].

There is little recent data regarding the risk of ICH in ADPKD patients receiving long-term renal replacement therapy. The objective of this study is to delineate differences in risk of ICH among the ADPKD vs. non-ADPKD patients by analyzing the United States Renal Data System (USRDS) database.

## Methods

### Patients and sources

We conducted a retrospective cohort study using the USRDS database. Because the identity of individual subjects is not disclosed and not obtainable from the USRDS datasets, and due to the substanial number of cases studied, individual consent was not obtained and the study was approved as exempt review by the Walter Reed National Military Medical Center Institutional Review Board. The variables included in the USRDS standard analysis files (SAFs), as well as data collection methods and validation studies, are listed at the USRDS website, under ‘Researcher’s Guide to the USRDS Database’, Section E, ‘Contents of all the SAFs’ (Standard Analysis Files), and published in the USRDS (http://www.usrds.org). The demographics of the dialysis population have been previously described [24]. The files SAF.PATIENTS were used as the primary dataset, including primary disease causing ESRD (PDIS) and cause and date of patient death. We defined the ADPKD cohort using the code (75313) for “polycystic kidneys, adult type (dominant)” from the PDIS variable. SAF.MEDEVID was used for additional information coded in the Medical Evidence Form starting in 1995, modified in 2005, and has highest sensitivity and specificity for cardiovascular outcomes [25]. We included patients who were initiated on chronic dialysis or transplanted from 1 January 1999 to 3 July 2009, followed until 31 Dec 2009 for Medicare claims of ICH or 2 December 2010 for outcomes of death (since Medicare related claims lag a year behind ascertainment of death). Study eligibility was restricted to patients with complete identifying and demographic information as well as evidence of Medicare as primary payer from the date of starting ESRD, as indicated from the PAYHIST file. This restriction was necessary in order to ensure accurate ascertainment of Medicare claims, which might not be reported in patients covered primarily by an insurer other than Medicare. Because ADPKD patients are significantly younger (mean age 54 years per USRDS report above) than patients with other causes of ESRD (mean age 64 years), restriction of our cohort to those with Medicare primary insurance, resulted in our including only 27% of total patients with ADPKD.

### Outcome variables

Our primary outcome, the incidence of ICH, was based on assessment of Medicare claims (either one inpatient claim or at least two outpatient claims) for any of the following ICD9 codes: 1) 430 “subarachnoid hemorrhage” 2) 432 “other and unspecified intracranial hemorrhage”. More specifically, ICD 430 included meningeal hemorrhage, ruptured berry aneurysm, and congenital aneurysm not otherwise specified (NOS), and excluded syphilitic cerebral aneurysm (094.87). ICD 432 included non-traumatic extradural/epidural hemorrhage (432.0), subdural hemorrhage (SDH) (432.1), and unspecified intracranial hemorrhage (432.9). We used diagnosis 1 and 2 as a composite outcome. The secondary outcome was all-cause mortality.

### Other predictor variables

Covariates included age at initiation of dialysis, gender, race (AA vs. non-AA), diabetes as a co-morbid condition, dialysis modality (for at least 60 days, from the treatment history files), body mass index, serum albumin and other co-morbid conditions from the Medical Evidence Form known to be associated with the outcomes, particularly prior history of stroke. Primary analysis included patients with prior history of cerebrovascular disease (cerebrovascular accident and transient ischemic attack) as a covariate. Furthermore, sensitivity analysis was conducted in a cohort of patients without a prior history of cerebrovascular disease.

### Statistical analysis

Analyses were performed using Stata 12.1 SE (Stata Corp, College Station, TX). Chi-square test (with Fisher exact test used for instances of fewer than 5 subjects) was used for assessment of categorical outcomes, Student’s t-test used for continuous outcomes, and non-parametric tests (Kruskal-Wallis) used for non-normal distributions. Kaplan-Meier analysis was used for unadjusted assessment of time to events. Cox regression was used for assessment of factors associated with ICH and mortality. Both graphical and formal methods were used to test the proportional hazards assumption. Model building used “forced” entry to account for factors known to be associated with outcomes based on existing literature, to avoid exclusion of such factors in stepwise models. Because ICH was a nonfatal outcome and could be precluded by the occurrence of death, death was also modeled as a competing risk in competing risk regression. Receipt of kidney transplant did not preclude the occurrence of intracranial hemorrhage but certainly could modify its occurrence due to selection and other factors. It was modeled both as a competing event in competing risk regression and a segmented time-dependent covariate in Cox regression (not modeled as a segmented time-dependent covariate in competing risks regression as this was too computationally intensive for our population size). Missing values for the variables serum albumin and body mass index (BMI, in kg/m^2^) were addressed by multiple imputation via linear regression method (STATA 12.1 multiple imputation module).

## Results

We identified 512,772 Medicare primary patients in the USRDS who were initiated on chronic dialysis or kidney transplantation between January 1, 1999 and July 2009, followed until 31 December 2009. Of these, a total of 8,793 patients (1.71%) developed ICH during the study period, and 6,749 (1.32% of total, or 1.42% of patients with non-missing causes of ESRD) had ADPKD as the primary cause of ESRD. For 2007 and 2008, our primary Medicare cohort included 27% and 26% respectively of all ESRD patients reported to the USRDS who had a primary diagnosis of ADPKD. Our primary Medicare cohort included 44% of all ESRD patients reported to the USRDS during that time. Thus, as expected, our primary Medicare cohort patients were significantly older than the total cohort, and less likely to have a primary diagnosis of ADPKD. Other demographic distinctions were not significant except for a higher percentage of black race.

Table 1 shows the unadjusted characteristics of patients in the ADPKD and non-ADPKD cohort. As compared to other causes of ESRD, patients with ADPKD were younger at start of dialysis and were more likely to be on peritoneal dialysis and to have received a kidney transplant. Other factors that were significantly associated with ADPKD included hypertension, tobacco use, higher mean hemoglobin, higher mean serum albumin, higher mean total cholesterol, higher mean low density lipoprotein (LDL) and BMI. Factors associated with non-ADPKD cohort were AA race, congestive heart failure, atherosclerotic heart disease, cerebrovascular disease, peripheral vascular disease, diabetes mellitus, chronic obstructive pulmonary disease, and alcohol dependence.

**Table 1:** Baseline demographic and comorbidity characteristics of study cohort from January 1999 to July 2009

**Variables**	**ADPKD**	**Non-ADPKD**	**P-value**
	**(n = 6,749)**	**(n = 506,023)**	
Gender			0.426
Male	3,551 (52.6)	268,709 (53.1)	
Female	3,198 (47.4)	237,314 (46.9)	
Race			<0.001
AA	848 (12.6)	123,256 (24.4)	
Non-AA	5,901 (87.4)	382,767 (75.6)	
Mean age at start of dialysis (yr)	65.30	70.20	< 0.001
	(95% CI 65.01-65.64)	(95% CI 70.21-70.28)	
Dialysis modality			<0.001
Hemodialysis	4,636 (79.4)	412,185 (92.4)	
Peritoneal dialysis	1,206 (20.6)	34,163 (7.7)	
Receipt of kidney transplant	1,784 (26.4)	24,743 (4.9)	<0.001
CHF	1,002 (14.9)	197,017 (39.6)	<0.001
Atherosclerotic heart disease	1,144 (17.0)	152,623 (30.7)	<0.001
Cerebrovascular disease (CVA, TIA)	541 (8.0)	59,859 (12.0)	<0.001
Peripheral vascular disease	452 (6.7)	91,337 (18.4)	<0.001
Hypertension	5,682 (84.4)	405,168 (81.4)	<0.001
Diabetes mellitus	480 (7.1)	212,998 (42.8)	<0.001
COPD	470 (7.0)	55,199 (11.1)	<0.001
Tobacco use	426 (6.3)	23,207 (4.7)	<0.001
Alcohol dependence	43 (0.6)	4,862 (1.0)	0.005
Mean hemoglobin (g/dL)	10.81	10.16	<0.001
	(95% CI 10.75-10.87)	(95% CI 10.16-10.17)	
Mean serum albumin (g/dL)	3.66	3.13	<0.001
	(95% CI 3.63-3.69)	(95% CI 3.13-3.14)	
Mean total cholesterol (mg/dL)	159.50	151.16	<0.001
	(95% CI 155.86-163.14)	(95% CI 150.68-151.64)	
Mean low density lipoprotein (mg/dL)	92.61	87.41	0.017
	(95% CI 88.39-96.83)	(95% CI 86.85-87.96)	
Mean body mass index (kg/m2)	27.10	28.34	<0.001
	(95% CI 26.83-27.37)	(95% CI 28.30-28.37)	

Data are n (%) or mean ± standard deviation.

Univariate analyses were performed with Chi-square testing for categorical variables (Fisher’s exact test used for violations of Cochran’s assumptions) and Student's *t*-test for continuous variables (Mann–Whitney test used for non-normally distributed variables).

ADPKD: autosomal dominant polycystic kidney disease; AA: African American; COPD: chronic obstructive pulmonary disease; CHF: congestive heart failure; CVA: cerebrovascular accident; TIA: transient ischemic attack.

**Figure 1:**
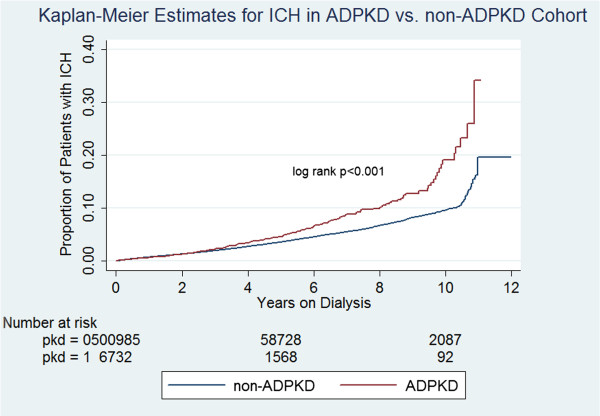
**Proportion of patients starting chronic dialysis therapy in the United States who developed intracranial hemorrhage (ICH), starting with the first date of dialysis.** Patients with autosomal dominant polycystic kidney disease (ADPKD) as cause of the end stage kidney disease had a significantly higher incidence of ICH, especially after the third year on dialysis.

**Figure 2:**
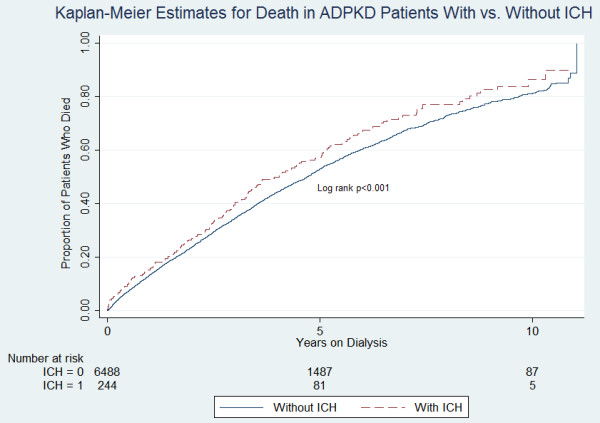
**Proportion of patients with ADPKD who died, comparing those who did not experience ICH (starting at the date of first dialysis) with those who experienced ICH (starting with the date of ICH).** Those with ICH had significantly higher subsequent risk of death.

Unadjusted analyses showed that 3.67% of ADPKD patients developed ICH vs 1.96% of non-ADPKD patients (p < 0.001) (Figure 1). ADPKD and non-ADPKD patients had 10.93 (95% CI 9.64-12.39) and 7.51 (95% CI 7.35-7.67) episodes of ICH per 1000 PY, respectively. For every 51.5 patients with ADPKD, there was one additional case of ICH, compared to patients without ADPKD (95% CI, 41.9-67.0). 0.71% of ADPKD patients had ruptured berry aneurysm vs 0.33% of non-ADPKD patients (p < 0.001). The mean time to ruptured berry aneurysm was 3.70 years from initiation of chronic dialysis in ADPKD patients vs 2.36 years for non-ADPKD patients (p < 0.001). All-cause mortality was 45.41% among ADPKD patients vs 71.89% among non-ADPKD patients (p < 0.001). Among the ADPKD cohort, those patients with ICH had a cumulative mortality risk of 64.08% vs. 44.71% among those without ICH (p <0.001) (Figure 2). Among the non-ADPKD cohort, those patients with ICH had a higher mortality risk of 82.42% vs 71.71% among those without ICH (p <0.001).

Table 2 shows the results of the adjusted Cox regression analysis of the factors associated with development of ICH. ADPKD was a significant predictor for ICH with an adjusted hazard ratio (AHR) of 1.63. After exclusion of patients who had a history of cerebrovascular disease including cerebrovascular accident (CVA) and transient ischemic attack (TIA), the risk of ICH among ADPKD patients still remained significant with an AHR 1.59 (95% CI 1.39-1.84). Other significant risk factors included age, year of first ESRD service, male gender and AA race. Furthermore, hemodialysis represented a significant risk factor for ICH as compared to peritoneal dialysis (AHR 1.32). Diabetes mellitus, hypertension, atherosclerotic heart disease, congestive heart failure, peripheral vascular disease, chronic obstructive pulmonary disease tobacco use and alcohol dependence were not significantly associated with ICH.

**Table 2:** Cox proportional hazard model to evaluate for predictive variables of intracranial hemorrhage

**Variables**	**Adjusted hazard ratio**	**P-value**	**95% Confidence interval**
ADPKD (vs. non-ADPKD)	1.63	<0.001	1.42-1.86
Hemodialysis vs peritoneal dialysis	1.32	<0.001	1.21-1.45
Age at start of dialysis	1.02	<0.001	1.02-1.02
Year of starting dialysis (vs. more recent year)	1.07	<0.001	1.06-1.08
Gender (male vs female)	1.05	0.04	1.00-1.09
Race (AA vs non-AA)	1.06	0.015	1.01-1.12
Diabetes mellitus	0.99	0.717	0.95-1.04
Alcohol dependence	1.10	0.412	0.87-1.40
Tobacco use	1.01	0.831	0.90-1.13
Chronic obstructive pulmonary disease	0.94	0.127	0.87-1.02
Hypertension	0.95	0.109	0.90-1.01
Atherosclerotic heart disease	0.97	0.207	0.92-1.02
Congestive heart failure	1.04	0.099	0.99-1.09
Peripheral vascular disease	1.01	0.786	0.95-1.07
Cerebrovascular disease (CVA, TIA)	1.46	<0.001	1.38-1.56

ADPKD: autosomal dominant polycystic kidney disease; AA: African-American CVA: cerebrovascular accident; TIA: transient ischemic attack.

In a separate Cox model, we included imputed variables of serum albumin and BMI as covariates, given significant missing values (68.7% of serum albumin values and 60.3% of BMI values missing). With the imputed serum albumin as a covariate, ADPKD had an AHR 1.64 (95% CI 1.43-1.87) for ICH. In a similar analysis with the imputed BMI as a covariate, ADPKD had an AHR 1.62 (95% CI 1.42-1.85) for ICH.

Given that kidney transplant and death represented competing risks for the development of ICH, we performed competing risk regression which demonstrated that ADPKD had a subhazard ratio of 2.97 for ICH (vs. non-ADPKD) (Table 3). Furthermore, receipt of a kidney transplant, when modeled as a time-dependent covariate in Cox regression analysis, demonstrated an AHR of 0.77 for ICH in ADPKD patients during the post-transplant period (vs. pre-transplant period) (95% CI 0.67-0.89). Other significant factors for ICH were AA race, hemodialysis vs peritoneal dialysis, year of starting dialysis vs more recent year, and hypertension.

**Table 3:** Competing risks regression model to evaluate for predictive variables of intracranial hemorrhage, with death and kidney transplant as competing risks

**Variables**	**Adjusted subhazard ratio**	**P-value**	**95% Confidence interval**
ADPKD (vs. non-ADPKD)	2.97	<0.001	2.27-3.89
Age at start of dialysis	0.98	<0.001	0.98-0.99
Gender (male vs. female)	0.99	0.952	0.89-1.11
Race (AA vs. non-AA)	1.29	<0.001	1.14-1.46
Hemodialysis vs peritoneal dialysis	1.64	<0.001	1.29-2.07
Year of starting dialysis (vs more recent year)	1.39	<0.001	1.36-1.42
Diabetes mellitus	0.76	<0.001	0.68-0.85
COPD	0.66	<0.001	0.54-0.82
Hypertension	1.23	0.008	1.06-1.43
Congestive heart failure	0.79	<0.001	0.69-0.89
Atherosclerotic heart disease	0.93	0.275	0.81-1.06
Peripheral vascular disease	0.85	0.053	0.72-1.00
Tobacco use	0.78	0.079	0.59-1.03
Alcohol dependence	0.89	0.679	0.50-1.58
Serum albumin < 3gm/dL	0.93	0.326	0.80-1.08
BMI < 20 kg/m2	0.80	0.091	0.63-1.04

ADPKD: autosomal dominant polycystic kidney disease; AA: African-American; COPD: chronic obstructive pulmonary disease; BMI: body mass index.

Adjusted Cox regression demonstrated that ADPKD patients had an AHR of death of 0.59 vs non-ADPKD patients (95% CI 0.57-0.61). AHRs for death among ADPKD patients were similar when Cox regression analyses were performed across specific age groups: AHR 0.45 (95% CI 0.31-0.64) for the 20–40 year-old group; AHR 0.49 (95% CI 0.45-0.55) for the 40–60 year-old group; AHR 0.57 (95% CI 0.55-0.60) for the 60–80 year-old group.

## Discussion

In our competing risk model, the ICH incidence was significantly higher in the ADPKD-patients vs. non-ADPKD-patients with an adjusted subhazard ratio of 2.97. Despite the significantly greater relative risk of ICH among ADPKD patients, however, their absolute risk of ICH (10.9/1000 PY) was still relatively low compared to non-ADPKD patients (7.5/1000 PY), resulting in a relatively high number of patients (29.4 using incidence density) who would need to be followed to observe one additional case of ICH, compared to patients without ADPKD. In adjusted Cox analysis, ADPKD was a significant predictor of ICH in ESRD patients on renal replacement therapy. We also observed that the increased risk of ICH associated with ADPKD did not manifest until approximately three years on dialysis. This could be related to the relatively older age of the ADPKD cohort (65.3 years at initiation of dialysis). Older age was a significant risk factor for ICH in our study and thus consistent with the finding of higher risk of ICH among ADPKD patients with accrued time on renal replacement therapy. Also, a significantly higher proportion of ADPKD patients received a kidney transplant compared to the non-ADPKD patients (26.4% vs. 4.9%, respectively), and based on our finding of lower risk of ICH post-transplant vs. pretransplant period, we may speculate that a kidney transplant had an initial mitigating effect on the risk of ICH among ADPKD patients.

ICH incidence increases with age, tobacco use, hypertension, heavy ethanol use, and female sex after age 60 [26]. However, there has been no large study comparing the incidence and risk factors for ICH in ADPKD patients vs. non-ADPKD patients on renal replacement therapy. In a previous USRDS study by Seliger et al., ADPKD was not reported as a significant risk factor for hemorrhagic stroke, with an AHR of 2.55 (p = 0.07) [27]. This study, conducted before 1997, was approximately 13,000 patients with 710 ADPKD patients and 910 strokes. For a relatively rare diagnosis, such as ADPKD, these findings are not inconsistent allowing for differences in sample size and methods. The Seliger study included important information on blood pressure at dialysis initiation, but did not use competing risks regression to treat death and kidney transplant as competing events.

The ADPKD cohort in our study had a lower mortality rate compared to the non-ADPKD cohort, even when age-matched. This is in contrast to a previous study by Pirson comparing an ADPKD vs. non-ADPKD cohort on hemodialysis in which there was no statistically significant difference in mortality [28]. However, the Pirson study was not sufficiently powered to detect a significant mortality difference, as there were only 50 patients in each arm. It is also possible that there is less risk for atherosclerotic cardiovascular disease among patients with ADPKD with ESRD compared to those with ESRD due to other etiologies. In our study, the non-ADPKD cohort had a higher prevalence of CHF, atherosclerotic heart disease, peripheral vascular disease, cerebrovascular accident, and had a higher BMI and lower serum albumin, all suggestive of a less healthy patient population compared to the ADPKD cohort.

There is a 35-55% combined case mortality and morbidity following intracerebral aneurysm rupture in ADPKD patients [12,29]. Not surprisingly, within our ADPKD cohort, those with ICH had a higher death rate compared to those without ICH.

AA race was a significant risk factor for ICH in our study. This is consistent with other literature where the incidence rate ratios relative to whites ranged from 1.4-1.8 among non-hemodialysis patients [30-32]. In the USRDS study by Seliger mentioned above, black patients on hemodialysis who did not have cardiovascular disease had twice the rate of hemorrhagic stroke compared to the white cohort [21].

ADPKD patients who underwent renal transplantation had a lower risk of ICH as compared to those who remained on dialysis, possibly reflecting pre-transplant screening and selection against or intervention in patients with aneurysms. ADPKD patients who were on hemodialysis had a higher risk of ICH compared to those who were on peritoneal dialysis. These findings may be explained by the blood pressure fluctuations associated with extracorporealization during hemodialysis, as well as required systemic anticoagulation. This is somewhat consistent with the USRDS study by Seliger mentioned above. In that study, receiving a transplant was associated with a lower risk of cerebrovascular event but there was no significant risk reduction with peritoneal dialysis compared to hemodialysis (did not differentiate between hemorrhagic stroke vs ischemic stroke) [21]. Another USRDS study which looked at subdural hematoma (ICD 432.1) showed a doubling incidence from 1991 to 2002 in the hemodialysis cohort but no significant upward trend for those on peritoneal dialysis [33]. This study again suggests the potential for intracranial hemorrhage due to blood pressure fluctuations and anticoagulation associated with hemodialysis.

About 19% of the ADPKD cohort who developed ICH had a known berry aneurysm, about double that of non-ADPKD cohort. A previous study of 129 ADPKD patients who died showed that 11% of them died due to ICH. Of these, about half had a ruptured intracranial aneurysm [34]. The prevalence of intracranial aneurysms might have been even higher than 19% among ADPKD patients who developed ICH, due to lack of screening. The prevalence of intracranial aneurysm in ADPKD is about 5-10% based on 3 prospective trials totaling 266 patients [11,35,36]. However, at least in the general population, the vast majority of intracranial aneurysms do not rupture [37] and therefore presumably do not need intervention, making a screening strategy based on detection of aneurysms problematic. Patients with PKD1 and PKD2 seem equally likely to develop intracranial aneurysms, while patients with mutations to the 5' half of PKD1 may more likely have vascular complications [38]. Screening has been reported and recommends focus on patients age <30, not applicable to the current study population [7].

### Limitations

There were several limitations with our study. Because our cohort was limited to Medicare primary recipients, which was required to strictly ascertain Medicare-defined outcomes, such as claims for specific diagnoses, the patients were significantly older than the USRDS population as a whole, and excluded a disproportionately higher fraction of patients with ADPKD. The USRDS does not have information on family history, which is a key determinant of further evaluation for cerebral aneurysm, especially with a known diagnosis of ADPKD. The USRDS also does not have information on ADPKD genotype, and we thus cannot distinguish between genotype PKD1 vs. genotype PKD2, which generally has a more indolent course than PKD1, ie later age at diagnosis, and later onset of hypertension and ESRD [39,40]. In any case, it may be even more impressive that a significant association was seen between ADPKD and subsequent ICH, as it is often assumed that ADPKD patients are past their high-risk period for intracranial events once they have started dialysis.

As a retrospective study, another limitation was the risk of misclassification. The primary outcome of ICH was based on ICD-9 codes which are entirely determined by physician documentation and standard clinical practice. Because all ADPKD patients are not screened for berry aneurysms, but only “high risk” patients, unscreened ADPKD patients may have an ICH due to intracranial aneurysm rupture, but may be coded as hemorrhagic CVA due to appearance on conventional imaging. Thus reliance solely on the diagnosis of subarachnoid hemorrhage could lead to under-ascertainment of the true incidence of ruptured intracranial aneurysms. Conversely, most hemorrhagic CVA’s in the general population or dialysis population are likely not related to ruptured intracranial aneurysm. This may not be the case for patients with ADPKD but the USRDS does not supply sufficient detail to definitively answer this question. Therefore, we performed sensitivity analyses of each outcome individually, in addition to the aggregate outcome, as used by previous investigators [11,20] and demonstrated similar findings. Due to the retrospective nature of our analysis, which assesses association only and not causality, we cannot account for screening and other surveillance biases. As noted previously, patients with ADPKD are more likely to be evaluated for intracranial aneurysms than patients with other causes of ESRD. However, the diagnosis of hemorrhagic CVA should not be susceptible to surveillance bias, which is why we included it as an outcome. In addition, information on family history was not available. The risk of ICH is likely restricted to a subgroup of all patients with ADPKD. However, this is essentially a type of residual confounding in which the risk of any categorical variable (ie, diabetes, hypertension) is not uniformly distributed among all subjects with the condition, but rather varies according to the severity of the condition. This is a common limitation of all registry data.

Demographically, for purposes of analysis, patients were considered to be African-American or not. As discussed earlier, our cohort was older compared to the mean age at the time of intracranial aneurysm rupture reported among ADPKD patients (65.3 years vs. 41 years).

Strengths of the study are its large size and consistency of findings over many years. The study describes national physician practice as documented by standard coding techniques. Single center studies, and even multi-center studies unless conducted over many years, would not be large enough to assess differences in the outcomes that were assessed.

## Conclusions

Medicare primary patients with ESRD due to ADPKD were at independently increased risk of ICH compared to patients with other causes of ESRD. While the nature of our analysis cannot entirely exclude issues of misclassification and surveillance bias, our results strongly suggest that an increased risk of ICH continues for patients with ADPKD even after onset of dialysis. Peritoneal dialysis was associated with a lower risk of ICH which can be potentially explained by the significant blood pressure fluctuations during hemodialysis as well as use of anticoagulants. The significantly lower risk of ICH for ADPKD patients after kidney transplantation suggests either that such patients are carefully screened prior to transplant and/or that remaining on dialysis poses a greater risk for subsequent ICH.

## Competing interests

The authors declare no competing interests.

## Authors’ contributions

DY: the primary investigator and formulated the original study topic and design. He performed all analyses and manuscript writing under mentorship of the senior investigators. LYA oversees the infrastructure of the USRDS and participated in the writing of the manuscript. CMY mentored the primary investigator and made key contributions to the study design, and participated in writing of the manuscript. KCA made possible the analytical infrastructure for the study and oversaw all analyses, mentored the primary investigator, and participated in the writing of the manuscript. RN was the primary mentor for the primary investigator, performed all analyses and manuscript writing. All authors read and approved the final manuscript.

## Pre-publication history

The pre-publication history for this paper can be accessed here:

http://www.biomedcentral.com/1471-2369/15/39/prepub
